# Pituitary adenylate cyclase-activating polypeptide (PACAP) has a neuroprotective function in dopamine-based neurodegeneration in rat and snail parkinsonian models

**DOI:** 10.1242/dmm.027185

**Published:** 2017-02-01

**Authors:** Gabor Maasz, Zita Zrinyi, Dora Reglodi, Dora Petrovics, Adam Rivnyak, Tibor Kiss, Adel Jungling, Andrea Tamas, Zsolt Pirger

**Affiliations:** 1 MTA-ÖK BLI NAP_B Adaptive Neuroethology, Department of Experimental Zoology, Balaton Limnological Institute, MTA-CER, 8237 Tihany, Hungary; 2 Department of Analytical Biochemistry, Institute of Biochemistry and Medical Chemistry, University of Pecs, 7624 Pecs, Hungary; 3 Department of Anatomy, University of Pecs, 7624 Pecs, Hungary; 4 Department of Experimental Zoology, Balaton Limnological Institute, MTA-CER, 8237 Tihany, Hungary

**Keywords:** Rotenone, 6-OHDA, PD models, Dopamine, PARK7, DJ-1, PACAP

## Abstract

Pituitary adenylate cyclase-activating polypeptide (PACAP) rescues dopaminergic neurons from neurodegeneration and improves motor changes induced by 6-hydroxy-dopamine (6-OHDA) in rat parkinsonian models. Recently, we investigated the molecular background of the neuroprotective effect of PACAP in dopamine (DA)-based neurodegeneration using rotenone-induced snail and 6-OHDA-induced rat models of Parkinson's disease. Behavioural activity, monoamine (DA and serotonin), metabolic enzyme (S-COMT, MB-COMT and MAO-B) and PARK7 protein concentrations were measured before and after PACAP treatment in both models. Locomotion and feeding activity were decreased in rotenone-treated snails, which corresponded well to findings obtained in 6-OHDA-induced rat experiments. PACAP was able to prevent the behavioural malfunctions caused by the toxins. Monoamine levels decreased in both models and the decreased DA level induced by toxins was attenuated by ∼50% in the PACAP-treated animals. In contrast, PACAP had no effect on the decreased serotonin (5HT) levels. S-COMT metabolic enzyme was also reduced but a protective effect of PACAP was not observed in either of the models. Following toxin treatment, a significant increase in MB-COMT was observed in both models and was restored to normal levels by PACAP. A decrease in PARK7 was also observed in both toxin-induced models; however, PACAP had a beneficial effect only on 6-OHDA-treated animals. The neuroprotective effect of PACAP in different animal models of Parkinson's disease is thus well correlated with neurotransmitter, enzyme and protein levels. The models successfully mimic several, but not all etiological properties of the disease, allowing us to study the mechanisms of neurodegeneration as well as testing new drugs. The rotenone and 6-OHDA rat and snail *in vivo* parkinsonian models offer an alternative method for investigation of the molecular mechanisms of neuroprotective agents, including PACAP.

## Abstract (precis)

**Summary:** PACAP has a neuroprotective effect in different toxin-induced rat and snail parkinsonian models, acting partially through the same mechanisms.

## INTRODUCTION

Parkinson's disease (PD) is characterized by dopaminergic (DAergic) neuron loss in the substantia nigra pars compacta (SNc) leading to progressive motor disability (Damier et al., 1999). Although several pharmacological compounds can mitigate motor and non-motor symptoms of the disease, the applied drugs are unable to cure or prevent it from progression (Schapira, 2005). The ideal therapy without long-term side effects is currently not available for patients suffering from PD; therefore, it is of the utmost importance to find or develop new drugs that prevent neurodegeneration and illness progression (LeWitt, 2016; Schapira et al., 2006).

Toxin-induced animal models have contributed substantially to the elucidation of the pathogenic mechanisms and the pathophysiology underlying PD. The most widely used parkinsonian models are generated by 6-hydroxy-dopamine (6-OHDA), rotenone, 1-methyl-4phenyl-1,2,3,6-tetrahydropyridine (MPTP), paraquat and amphetamine (Jagmag et al., 2016). These chemicals have all been shown to be neurotoxins that induce a loss of DAergic neurons in the SNc and ventral tegmental area (VTA) in model animals. Neurotoxins reproduce one or more key pathological features of clinical PD within a shorter period of time in animals. For example, both 6-OHDA and rotenone induce progressive DAergic neuronal degeneration by generating oxidative stress. It is observed that 6-OHDA affects mitochondrial translocation of the PARK7 (also known as DJ-1) chaperone protein (Miyama et al., 2011). In addition, rotenone also affects α-synuclein phosphorylation, inducing formation of Lewy-body- and ubiquitin-like inclusions (Bove and Perier, 2012; Jagmag et al., 2016; Sonia et al., 2013). Behavioural alterations and α-synuclein inclusions, however, reveal variations underlying the limited validity of different parkinsonian models (Bove and Perier, 2012). Nevertheless, rotenone is highly lipophilic and easily crosses the blood-brain barrier (BBB); therefore, it can be injected intraperitoneally, intravenously or subcutaneously for systemic treatment (Jagmag et al., 2016). There is only one publication where rotenone has been unilaterally injected into the rat brain (Xiong et al., 2009). Although rotenone induces loss of DAergic neurons, this model is difficult to replicate owing to the high mortality rate of the rats. The hydrophilic compound 6-OHDA (which cannot cross the BBB) is a generally used selective toxin to induce DAergic neuronal cell death by direct injection in the SNc or VTA in rats (Jagmag et al., 2016).

It has been found that rotenone-induced lesions also replicate some symptoms of PD in invertebrates (Penney and Mccabe, 2008). Chronic exposure to rotenone induces loss of tyrosine-hydroxylase immunoreactivity in the giant DAergic neuron (RPeD1) of the giant pond snail, suggesting that rotenone affects the DAergic system. Decrease of brain DA content results in a progressive and irreversible decline in locomotion, feeding and in the life span of treated snails (Vehovszky et al., 2007). The usefulness of modelling diseases in invertebrates is often questioned; however, physiological events at the molecular and cellular levels established a number of similarities between vertebrate and invertebrate PD models (Penney and Mccabe, 2008).

Since previous drugs have failed to successfully treat the disease in clinical trials, it is suggested that multi-pathway drugs may overcome this challenge. For that reason, we investigated the neuroprotective effect of pituitary adenylate cyclase-activating polypeptide (PACAP) to prevent DAergic neurons from degeneration in the 6-OHDA-induced rat and rotenone-induced snail parkinsonian models. PACAP belongs to the vasoactive intestinal peptide/secretin/glucagon peptide family and it acts through G-protein-coupled specific (PAC1-R) and unspecific (VPAC1-R, VPAC2-R) receptors (Reglodi et al., 2015). PACAP as neuropeptide is expressed widely in the brain of mammals at particularly high concentrations in the hypothalamus, substantia nigra (SN), nucleus accumbens and bed nucleus of the stria terminalis (Bourgault et al., 2009; Ghatei et al., 1993; Palkovits et al., 1995; Vaudry et al., 2009), as well as in different parts of the central nervous system (CNS) of Oligochaetes, gastropods and insects (Pirger et al., 2016). In numerous studies it has been shown that PACAP exerts pleiotropic effects on neuronal and non-neuronal cells, including neuroprotective effects (Reglodi et al., 2011, 2012, 2015). PACAP rescues DAergic neurons from neurodegeneration and improves motor alterations induced by unilateral 6-OHDA or MPTP injection in rat models of PD (Reglodi et al., 2004; Somogyvari-Vigh and Reglodi, 2004; Takei et al., 1998; Wang et al., 2008). Its anti-apoptotic effect has also been observed in an invertebrate animal (Pirger et al., 2008).

Comparison of different parkinsonian models at the molecular level may provide new information about the pathological mechanisms of the disease and open new avenues in the development of novel anti-neurodegenerative and neuroprotective approaches. Our main question was whether the neuroprotective effect of PACAP correlated with changes observed at the molecular and system levels in the 6-OHDA and rotenone toxin models. We investigated the effect of exogenous PACAP on the behavioural activity (feeding and locomotion), level of monoamines [dopamine (DA) and serotonin (5HT)], DA-metabolizing enzymes (S-COMT, MB-COMT and MAO-B) and PARK7 protein in rat and snail parkinsonian models *in vivo*. In our experiments, the widely used hydrophilic and selective agent 6-OHDA was applied to induce DAergic neuronal cell death in the rat model, while the highly lipophilic compound rotenone was used in the snail model.

## RESULTS

### DA and 5HT quantification

**Fig. 1.**
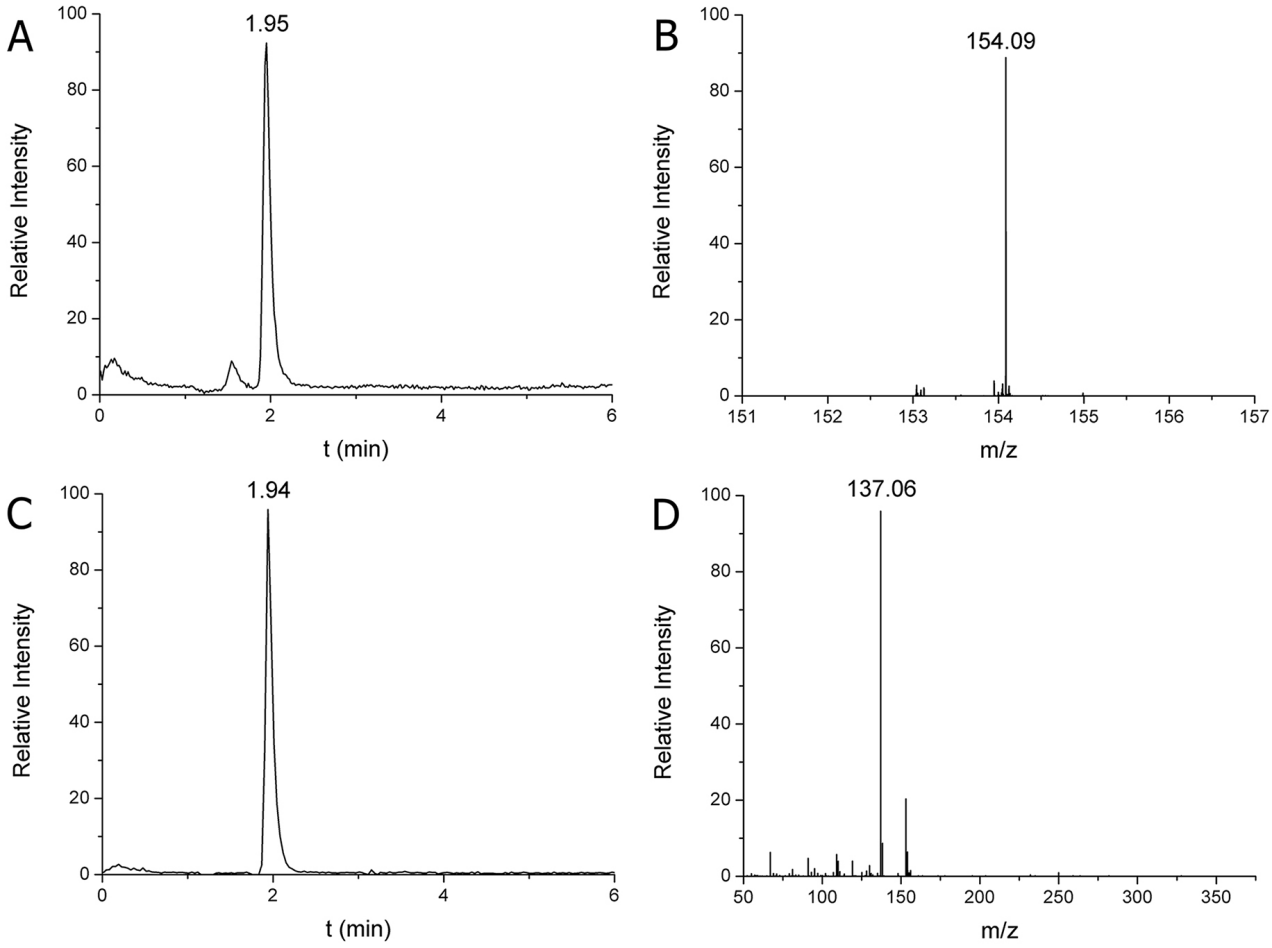
**HPLC-MS analysis of DA in SN of the rat.** (A) Extracted ion chromatogram of DA at 1.95 min retention time in single ion monitoring (SIM) mode. (B) MS spectrum of DA identified at 154.09 m/z parent ion protonated form [M+H]^+^. (C) Extracted ion chromatogram of DA fragment at 1.94 min retention time in MS/MS mode. (D) MS/MS spectrum of DA identified at 137.06 m/z fragment ion protonated form [F+H]^+^ (219 fmol DA was injected). The calculated DA content was 3.87 µg/g SN tissue.

**Fig. 2.**
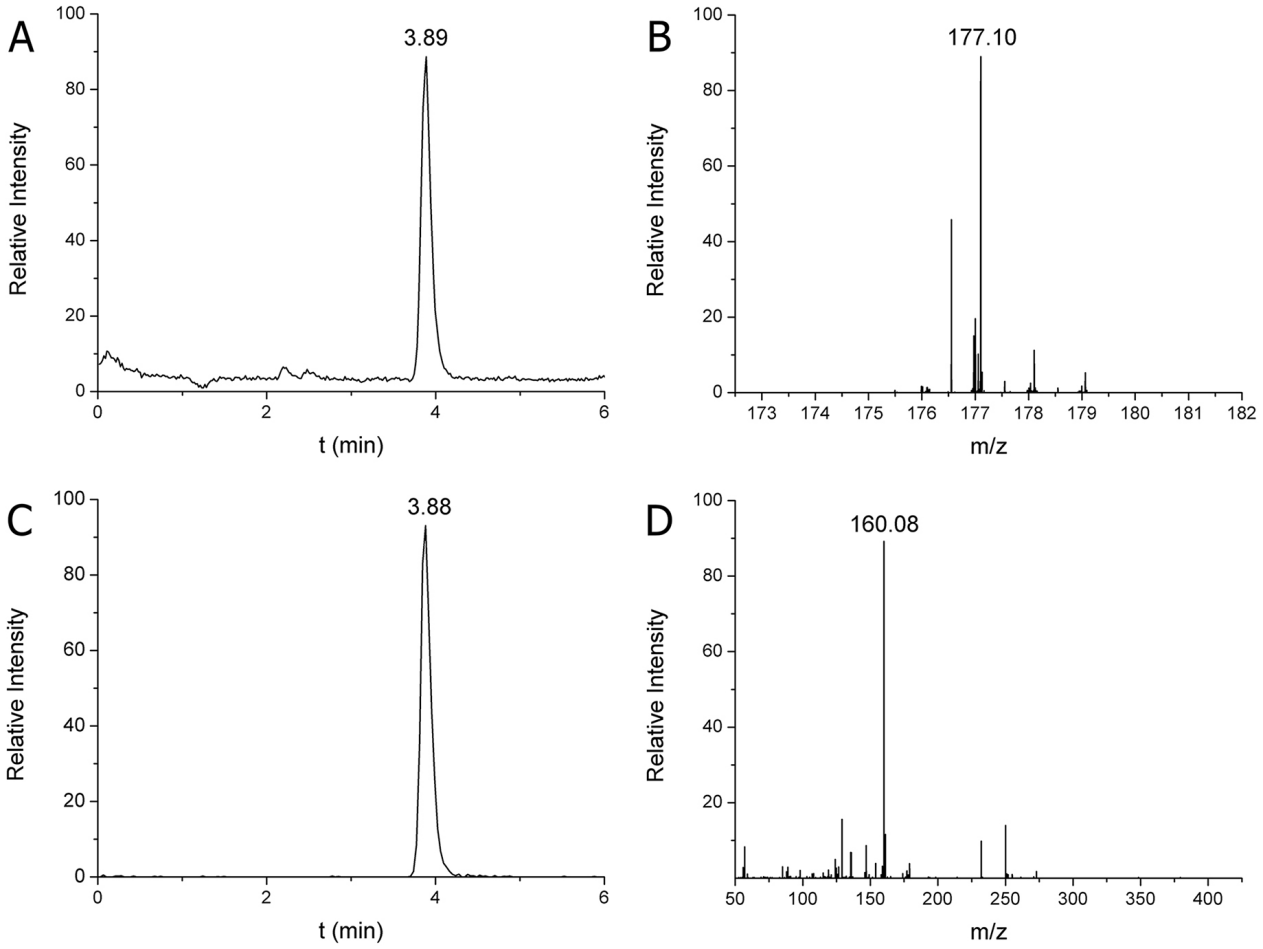
**HPLC-MS analysis of 5HT in SN of the rat.** (A) Extracted ion chromatogram of 5HT at 3.89 min retention time in single ion monitoring (SIM) mode. (B) MS spectrum of 5HT identified at 177.10 m/z parent ion protonated form [M+H]^+^. (C) Extracted ion chromatogram of 5HT fragment at 3.88 min retention time in MS/MS mode. (D) MS/MS spectrum of 5HT identified at 160.08 m/z fragment ion protonated form [F+H]^+^ (143 fmol DA was injected). The calculated 5HT content was 3.35 µg/g SN tissue.

Calibration curves were produced for the quantitative analysis of DA and 5HT, using 55, 111, 556, 834 and 1112 pmol/ml DA and 122, 305, 610, 1200 and 2100 pmol/ml 5HT as external standards. Correlation coefficients (*r*^2^) were between 0.9956 and 0.9989 for all acceptable calibration curves both in parent ion scan (MS) and fragment ion scan (MS/MS) modes (not shown). The limits of detection and quantification were 2.9 and 5.8 pmol/ml for DA, and 6.2 and 9.4 pmol/ml for 5HT, respectively. Based on own exact molecular weights and fragments, the structure of DA and 5HT were confirmed from rat SN and snail CNS homogenates. Quantification was performed parallel in both MS and MS/MS modes. DA was identified at 154.09 m/z parent ion protonated form and at 137.06 m/z fragment ion protonated form, which were characterized by a 1.94 min retention time (Fig. 1). The 5HT was identified at 177.10 m/z parent ion protonated form and at 160.08 m/z fragment ion protonated form, which were characterized by a 3.89 min retention time (Fig. 2). The identification and quantification parameters obtained from snail CNS homogenates exactly correspond with those of rat SN, therefore, they are not shown. The parent ion exact masses of DA and 5HT as well as their MS/MS transitions data confirm those described earlier (Wei et al., 2014). Based on these data, identification of monoamines both in invertebrate and vertebrate samples was successful.

### Effect of PACAP in an invertebrate model: rotenone-induced injury in molluscs

**Fig. 3.**
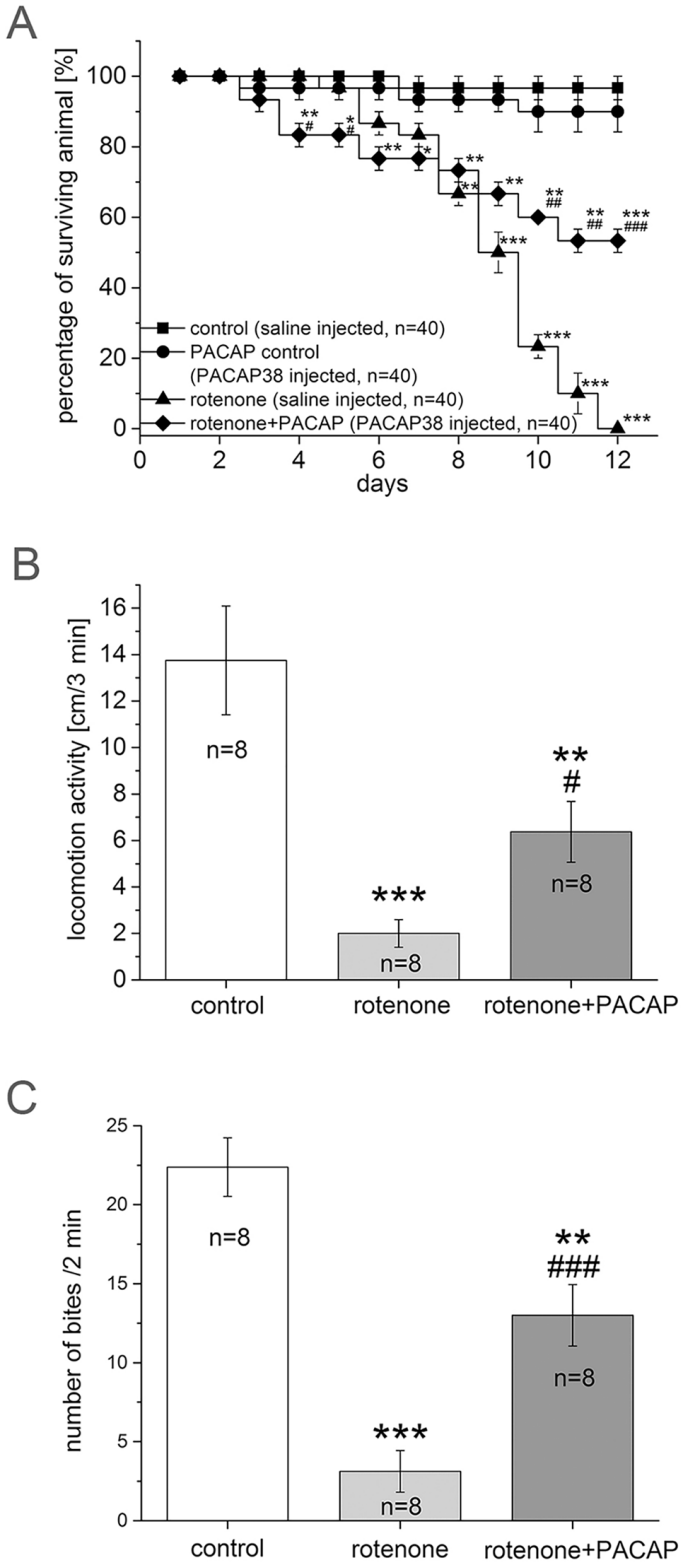
**Survival, locomotion and feeding of snails after treatment with PACAP, rotenone or rotenone+PACAP.** (A) Survival in controls and after treatment with PACAP, rotenone or rotenone+PACAP for 12 days. The total number of animals was *n*=40 from 4 independent experiments in each group. (B) Locomotor activity of snails of control (*n*=8), rotenone (*n*=8) and rotenone+PACAP (*n*=8) groups. Mean distances covered during the 3 min test period by snails are shown. One-way ANOVA: *F*_(2)_=13.99, *P*<0.001; *post hoc* test between control and rotenone groups (*P*<0.001), control and rotenone+PACAP (*P*<0.01); rotenone and rotenone+PACAP (*P*<0.05). (C) Feeding activity in snails of control (*n*=8), rotenone (*n*=8) and rotenone+PACAP (*n*=8) groups. Mean number of bites counted during 2 min time window are shown. One-way ANOVA: *F*_(2)_=32.66, *P*<0.001; *post hoc* test between control and rotenone (*P*<0.001), control and rotenone+PACAP (*P*<0.01); rotenone and rotenone+PACAP groups (*P*<0.001) (C). *P<0.05, **P<0.01, ***P<0.001 between control and treated groups; ^#^*P*<0.05, ^##^*P*<0.01, ^###^*P*<0.001 between rotenone and rotenone+PACAP groups.

The neuroprotective effect of PACAP in molluscs was studied in a rotenone-induced parkinsonian model. First, the physiological effect of PACAP, rotenone and rotenone+PACAP together was observed in a survival experiment (Fig. 3A). The average percentage of surviving snails was 97.5% in the control and 90% in the PACAP control groups. Although animals started to die from day 3-4 in the rotenone+PACAP group compared with day 5 in the rotenone group, snails treated with rotenone+PACAP survived significantly longer than those treated only with rotenone. Approximately 50% of the animals remained alive on day 12 in the rotenone+PACAP group (Fig. 3A), whereas all animals in the rotenone group died by day 12.

The spontaneous locomotor activity of both rotenone and rotenone+PACAP groups significantly decreased in the behavioural test on day 5 of treatment compared with control animals (Fig. 3B). The snails in the rotenone group covered an average distance of 2.0±0.5 cm in 3 min, while the control snails covered 13.75±2.34 cm in 3 min, representing an ∼85% (*P*<0.001) decrease in the locomotor activity. In contrast, the animals in the rotenone+PACAP group covered 6.37±1.30 cm in 3 min, which is only an ∼50% (*P*<0.01) decrease compared with the control group. The locomotion test shows a significant difference between the rotenone and the rotenone+PACAP groups (∼35%, *P*<0.05), indicating that PACAP was able to prevent the severe behavioural effect caused by rotenone in snails.

Rotenone treatment also caused a marked reduction in the feeding rate (Fig. 3C). The number of bites made by snails in the rotenone group on day 5 of treatment was 3.12±1.31 bites in 2 min, which is an ∼85% (*P*<0.001) decrease compared with the number of bites in control snails (22.37±1.85 bites/2 min). As in the locomotion test, the rotenone+PACAP group showed a significantly higher rate of feeding (13.00±1.94 bites/2 min) than snails in the rotenone group, resulting in a 40% (*P*<0.001) decrease compared with the control group (Fig. 3C).

**Fig. 4.**
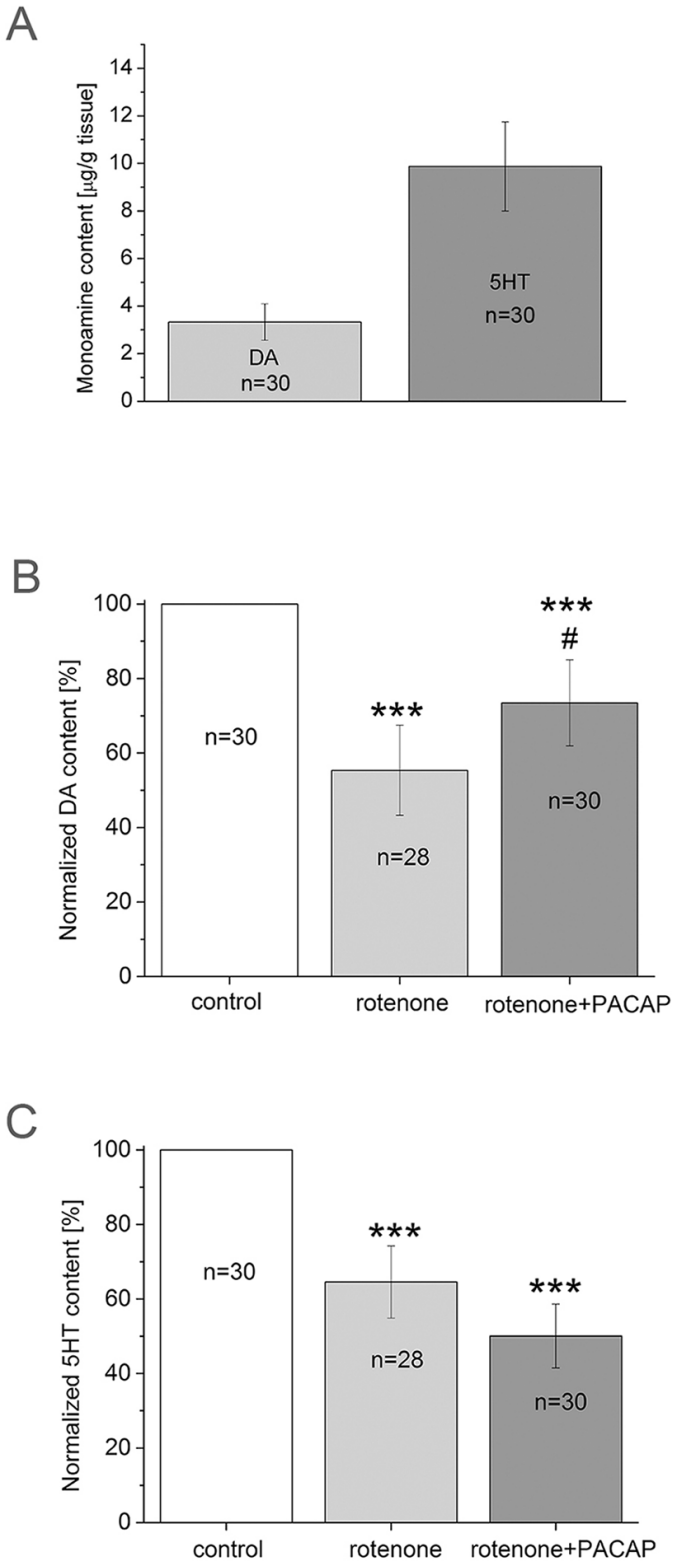
**Monoamine concentrations in snails treated with rotenone and rotenone+PACAP.** (A) Monoamine concentration in snail control group in µg/g tissue. (B) DA content as a percentage of control (*n*=30) in rotenone (*n*=28) and rotenone+PACAP (*n*=30) groups. One-way ANOVA, *F*_(2,86)_=39.03, *P*<0.001; *post hoc* test between control and rotenone groups (*P*<0.001), control and rotenone+PACAP groups (*P*<0.001); rotenone and rotenone+PACAP groups (*P*<0.05). (C) 5HT content as a percentage of control (*n*=30) in rotenone (*n*=28) and rotenone+PACAP (*n*=30) groups. One-way ANOVA, *F*_(2,86)_=18.41, *P*<0.001; *post hoc* test between control and rotenone (*P*<0.001), control and rotenone+PACAP (*P*<0.001). Total number of animals per group represents 3 independent experiments. ***P<0.001 between control and treated groups; ^#^*P*<0.05 between rotenone and rotenone+PACAP groups.

Since monoamines are relevant in locomotor activity during cilia movement and as they are also the main neurotransmitters in feeding musculature, we aimed to analyse the effect of PACAP on CNS monoamine levels. The average concentration of monoamines was measured using HPLC-MS in the CNS of control animals. The DA and 5HT content was found to be 3.33±0.76 and 9.87±1.87 µg/g tissue, respectively (Fig. 4A). These data correspond well to findings obtained earlier by HLPC-electrochemical detector (Nagy and Hiripi, 2002). Upon rotenone treatment, the DA content of the CNS was reduced to 55.3±12.15% (*P*<0.001) (normalized to control data, see Fig. 4A). In the rotenone+PACAP group, the decrease in the DA level was less pronounced: it decreased to 73.5±11.5% (*P*<0.001) (Fig. 4B), suggesting a marked protective effect of PACAP in this model (*P*<0.05 between the rotenone and rotenone+PACAP group). In contrast, the 5HT content decreased in both rotenone and rotenone+PACAP groups, with no significant difference between them. The 5HT level in the rotenone group decreased to 64.5±9.70% (*P*<0.001) and in the rotenone+PACAP group decreased to 49.9±8.60% (*P*<0.001) of control values (Fig. 4C).

**Fig. 5.**
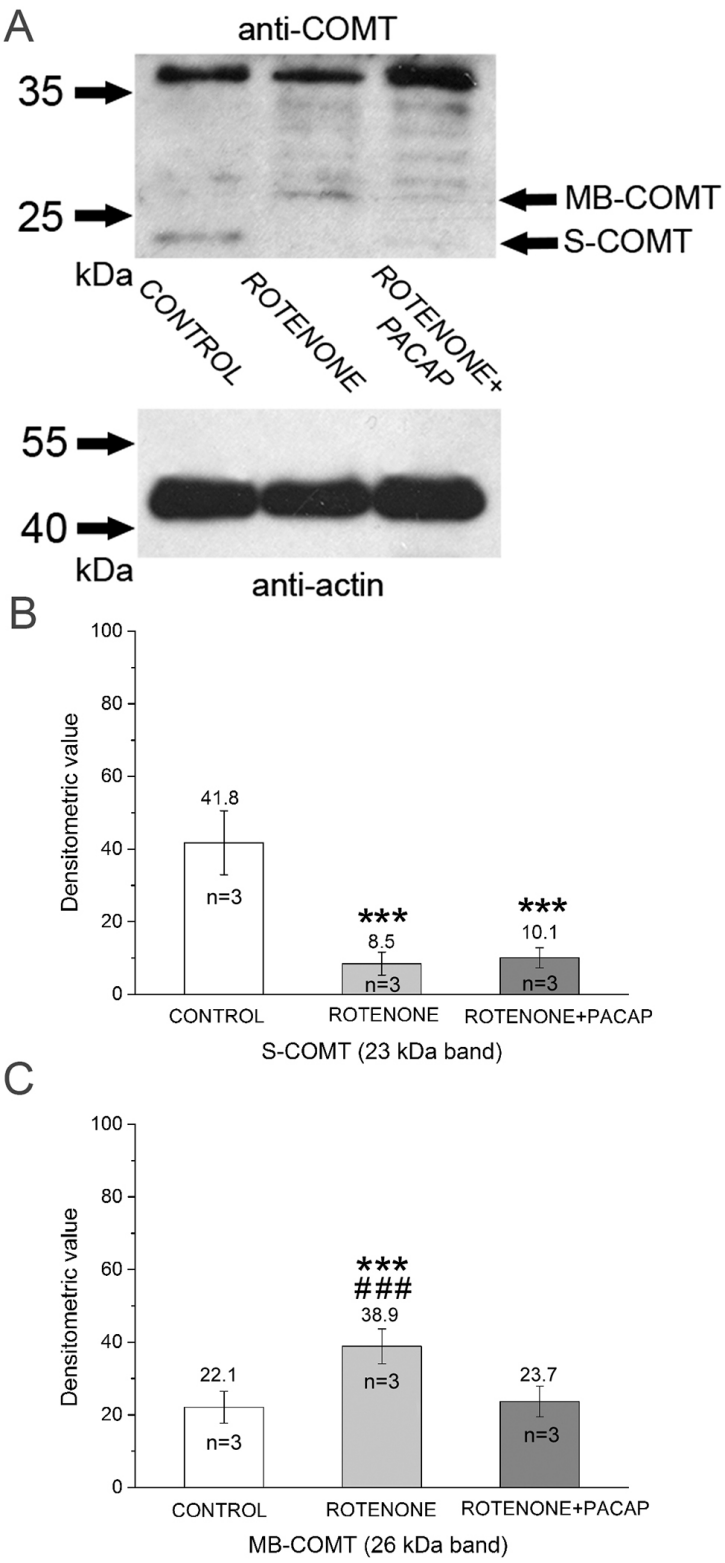
**Analysis of DA metabolic enzymes in snail brain homogenates.** (A) Western blot with anti-COMT. The bottom panel shows detection of anti-β-actin as a loading control. The top panel shows S- and MB-COMT localization at ∼23 and 26 kDa. A non-linear adjustment (+0.5% level adjustment) was applied to the MB-COMT band in rotenone group. Densitometry evaluations of S-COMT (B) and MB-COMT (C) (*n*=3, calculated from independent WB experiments). S-COMT: *F*_(2)_=135.80, *P*<0.001; *post hoc* test between control and rotenone groups (*P*<0.001), control and rotenone+PACAP groups (*P*<0.001). MB-COMT: *F*_(2)_=50.68, *P*<0.001; *post hoc* test between control and rotenone (*P*<0.001), rotenone and rotenone+PACAP groups (*P*<0.001). ***P<0.001 between control and treated groups; ^###^*P*<0.001 between rotenone and rotenone+PACAP groups.

Next, the metabolic enzymes of DA signalling were examined in control, rotenone and rotenone+PACAP groups by western blot (WB). According to Sloley (2004), vertebrate-like MAO-B plays a negligible role in metabolizing monoamines in the CNS of snails. Therefore, we focused our experiments on the other enzyme, COMT. The two forms, COMT soluble (S) and membrane bound (MB), had variable expression levels in the rotenone and rotenone+PACAP groups. Bands with a positive immune reaction were obtained at ∼23 and 26 kDa, corresponding well to values provided by the manufacturer (Fig. 5A). The immunopositive band of the 23 kDa S-COMT showed higher intensity in the control group (*P*<0.001), while this band had very low intensity in the rotenone and rotenone+PACAP groups (Fig. 5B). The 26 kDa band of the MB-COMT showed an average higher intensity (*P*<0.001) in animals of the rotenone group compared with the control group (*P*<0.001). The densitometry evaluation of MB-COMT did not show any change in rotenone+PACAP group compared with control animals (Fig. 5C). An interesting observation was the intense band at around 36 kDa (Fig. 5A). This unspecific immunopositive band decreased after rotenone treatment and it increased in the rotenone+PACAP group compared with controls. We do not have direct evidence about this protein in snail CNS but based on literature data, we speculate that this crossreactive band might be a sulfotransferase-like protein.

### Effects of PACAP in a vertebrate model: 6-OHDA-induced injury in rats

**Fig. 6.**
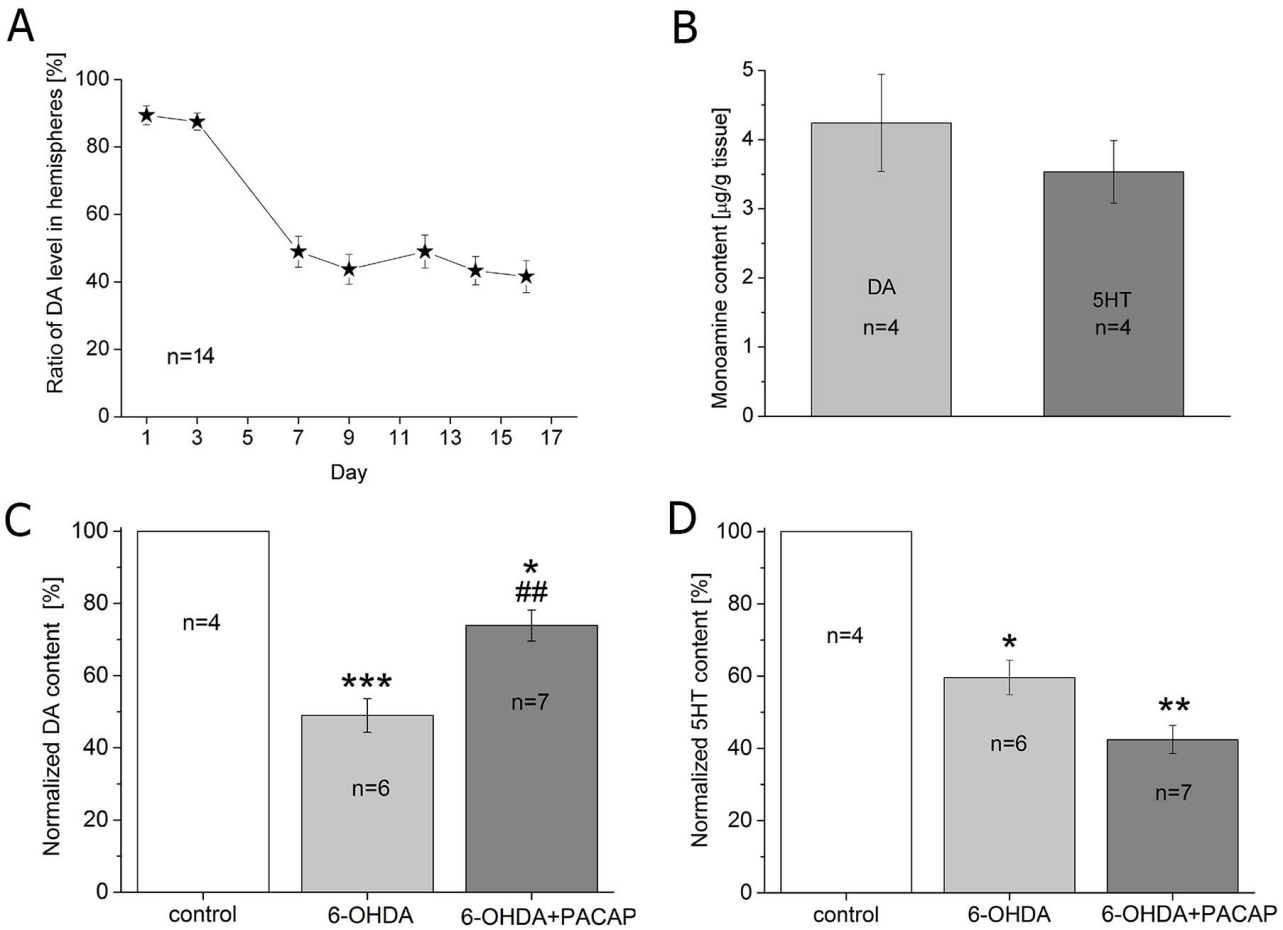
**Monoamine concentrations in rat SN after treatment with 6-OHDA.** (A) DA content of the SN 1, 3, 7, 9, 12, 14 and 16 days after operation (*n*=14). The *y*-axis represents the ratio of DA levels in the hemispheres. Data were calculated using the following formula: 100−(c_t_/c_c_×100), where c_t_=DA concentration of treated SN (6-OHDA-injected, left hemisphere); c_c_=DA concentration of the control SN (right hemisphere). (B) Monoamine content (DA and 5HT) of control group presented in µg/g tissue. (C,D) DA and 5HT content as a percentage of control (*n*=4) in 6-OHDA (*n*=6) and 6-OHDA+PACAP (*n*=7) groups. One-way ANOVA, DA: *F*_(2,18)_=15.49, *P*<0.001; *post hoc* test between control and 6-OHDA groups (*P*<0.001), control and 6-OHDA+PACAP groups (*P*<0.05); 6-OHDA and 6-OHDA+PACAP groups (*P*<0.01). 5HT: *F*_(2,12)_=12.87, *P*<0.001; *post hoc* test between control and 6-OHDA groups (*P*<0.05), control and 6-OHDA+PACAP groups (*P*<0.01) (D). *P<0.05, **P<0.01, ***P<0.001 between control and treated groups; ^##^*P*<0.01 between rotenone and rotenone+PACAP groups.

The ratio of DA content was determined from the SN 1, 3, 7, 9, 12, 14 and 16 days after 6-OHDA lesion. We observed that DA level continuously declined at the beginning and reached a maximum decrease on day 7 on the 6-OHDA-treated side compared with the control side. Thereafter, no significant decrease was observed (Fig. 6A). Therefore, for further experiments we sacrificed the animals on day 7 after the operation to measure the DA and 5HT levels of the SN. On the 7th postoperative day, the average DA level was 4.24±0.73 µg/g tissue, while the average 5HT level was 3.53±0.45 µg/g tissue in the SN of the control group (Fig. 6B). Following 6-OHDA treatment, levels of both DA and 5HT significantly decreased compared with control animals: the DA content of the SN was reduced to 48.71±4.65% (*P*<0.01) in the 6-OHDA-injected animals. PACAP treatment counteracted the 6-OHDA effect since DA content was reduced to only 73.93±4.31% (*P*<0.05) in the PACAP-treated group (Fig. 6C). A significant difference was observed between the 6-OHDA and 6-OHDA+PACAP groups (*P*<0.01). PACAP treatment did not cause a similar change in 5HT levels: its content in the SN was reduced to 59.64±4.77% (*P*<0.05) in 6-OHDA-treated animals and to 42.46±3.89% in 6-OHDA+PACAP-treated rats (*P*<0.01). A significant difference was not observed between the 6-OHDA and 6-OHDA+PACAP groups (Fig. 6D).

**Fig. 7.**
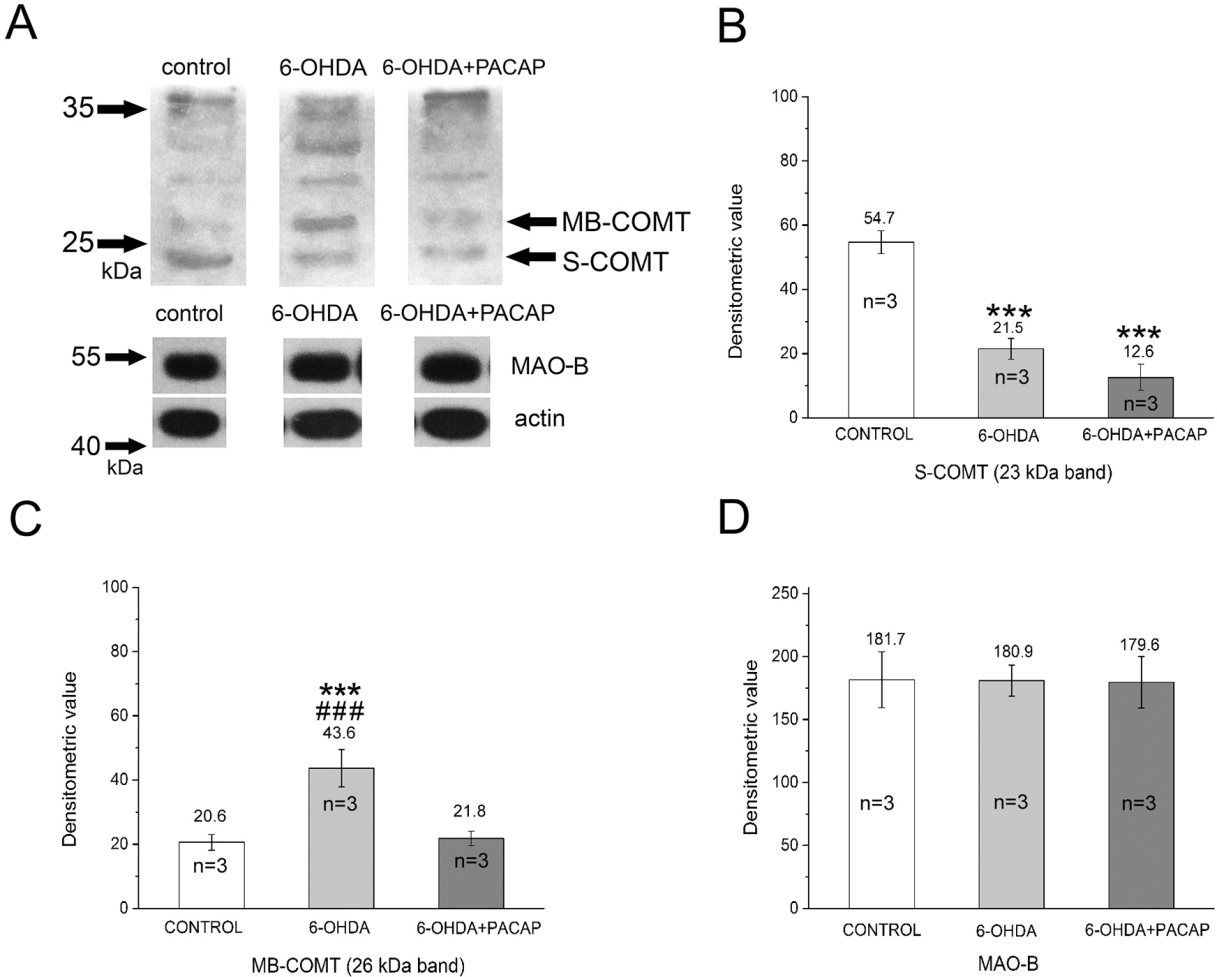
**Analysis of DA metabolic enzymes in rat SN after treatment with 6-OHDA.** (A) S- and MB-COMT at 23 and 26 kDa in control, 6-OHDA and 6-OHDA+PACAP groups are represented. Middle row shows the intensity of MAO-B. Anti-β-actin was used as an internal control (lower row). MAO-B (middle) and β-actin (bottom) levels were similar in all groups. Changes in concentration of S-COMT (B), MB-COMT (C) and MAO-B (D) detected by densitometry were identified at 23 kDa, 26 kDa and 55 kDa, respectively (*n*=3, calculated from independent WB experiments). S-COMT: *F*_(2)_=108.09; *P*<0.001; *post hoc* test between control and 6-OHDA groups (*P*<0.001), control and 6-OHDA+PACAP groups (*P*<0.001). MB-COMT: *F*_(2)_=134.42; *P*<0.001; *post hoc* test between control and 6-OHDA (*P*<0.001), 6-OHDA and 6-OHDA+PACAP groups (*P*<0.001). ***P<0.001 between control and treated groups; ^###^*P*<0.001 between 6-OHDA and 6-OHDA+PACAP groups.

Because PACAP had a protective effect on DA levels, in our further experiments we focused on DA metabolism only. We examined the possible changes in metabolic enzymes (MAO-B, COMT) of DA using WB (Fig. 7). Bands of 55 kDa were detected in control, 6-OHDA and 6-OHDA+PACAP groups by anti-MAO-B antibody (Fig. 7A). The total protein content of crude extract of SN was monitored using anti-β-actin (40 kDa) (Fig. 7A). The metabolic enzyme of DA was examined by anti-COMT antibody. Similar to invertebrate homogenates, the COMT was also identified in 23 and 26 kDa bands in rat samples, representing S-COMT and MB-COMT, respectively. The specific S-COMT band showed higher intensity in the control group, and significantly (*P*<0.001) decreased after 6-OHDA and 6-OHDA+PACAP treatment (Fig. 7B). The 26 kDa band of MB-COMT, however, showed different intensities between control, 6-OHDA and 6-OHDA+PACAP groups. The highest intensity of the MB-COMT was observed in the 6-OHDA group, while it was lower in the other two groups. Densitometric evaluation showed that the level of MB-COMT was significantly higher in the 6-OHDA group compared with the control or 6-OHDA+PACAP groups (*P*<0.001, Fig. 7C). Densitometry evaluation of the intensity of the positive MAO-B signal did not reveal any changes between the control and treated groups (Fig. 7D).

### Short-term neurotoxin effect at the proteomic level

Changes at the proteomic level have been described in the serum, SN and cerebrospinal fluid of PD patients and in the striatum of toxin-induced parkinsonian rats (Licker et al., 2014; Lu et al., 2014; Wang et al., 2013; Xiong et al., 2014). Therefore, we focused on the toxin-induced changes of protein composition and the possible protective effect of PACAP in the 6-OHDA model. Tissue homogenates of SN isolated from left (treated) and right (control) sides were separated by SDS-PAGE (*n*=3, Fig. S1). After separation, significant differences were not detected in the quality and quantity of total protein content on gel estimated by densitometry evaluation. Therefore, in order to determine the precise protein composition, the nanoLC-MS method was performed. Quantitative analysis of PD-relevant proteins (PARK7, myelin basic protein, D-dopachrome decarboxylase, thiosulfate sulfurtransferase) was applied to the 95 different proteins identified by database searching (Table S1). PARK7 protein, which belongs to the ThiJ/Pfp1/DJ-1 superfamily, presented a marked difference after treatment. This important chaperone protein was identified from the ∼20 kDa complex band, which also contained four other proteins (phosphatidylethanolamine-binding protein, UMP-CMP kinase, Ras-related protein Rab-6A, peroxiredoxin-1). PARK7 was the only identified protein in the control group that had different quantitative parameters, in contrast to other four proteins, which were present in all groups at similar amounts independent of treatment.

**Fig. 8.**
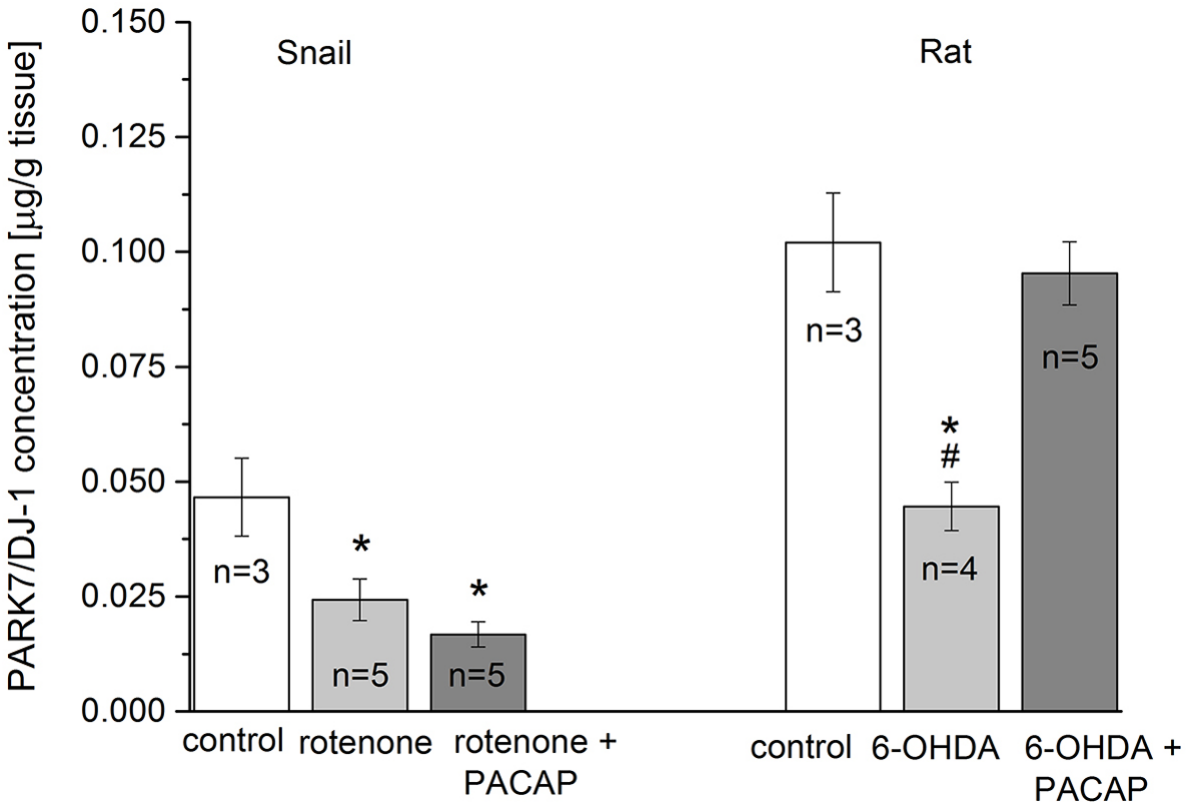
**Comparison of PARK7 levels in toxin-treated snail CNS and rat SN.** Values were measured in control (*n*=3) and after treatment with the different toxins (rotenone, *n*=5; 6-OHDA, *n*=4) and toxin+PACAP-treated groups (*n*=5). Concentration of PARK7 protein is given in μg/g unit. Snails: *F*_(2)_=24.59, *P*<0.05; *post hoc* test between control and rotenone groups (*P*<0.05), control and rotenone+PACAP groups (*P*<0.05). Rat: *F*_(2)_=6.90, *P*<0.05; *post hoc* test between control and 6-OHDA (*P*<0.05), 6-OHDA and 6-OHDA+PACAP groups (*P*<0.05). *P<0.05 between control and treated groups; ^#^*P*<0.05 between toxin and toxin+PACAP groups.

Finally, sandwich ELISA was used for the precise quantitative determination of PARK7 protein in both toxin models. Fig. 8 shows the PARK7 concentrations (μg/g) in snail and rat samples. In the rotenone-induced snail model, the PARK7 concentration significantly decreased (*P*<0.05) and could not be restored by PACAP (*P*<0.05). In the 6-OHDA-induced model, the level of PARK7 protein in the SN of the rat brain was significantly lower compared with the control level (*P*<0.05). However, in PACAP-pretreated rats, the PARK7 protein level was almost completely restored (*P*<0.05).

## DISCUSSION

### Effect of toxins on behaviour, monoamines, enzymes and PARK7 protein levels

The locomotion and rate of feeding activity were significantly decreased in rotenone-treated snails compared with levels in controls. The locomotion data correspond well to findings from rat experiments where parkinsonian symptoms are induced by 6-OHDA and the animals show hypokinetic behavioural signs in activity and asymmetrical measures (Tamas et al., 2006).

The DA and 5HT levels significantly decreased in both the 6-OHDA- and rotenone-induced models compared with the control hemisphere or control group. The decrease of monoamine levels correlated well with the degeneration of DAergic and 5HTergic systems, which provide the background for development of symptoms both in vertebrates and invertebrates (Duty and Jenner, 2011; Hely et al., 2005; Reglodi et al., 2004; Vehovszky et al., 2007). Recently, it has been suggested that 5HT-mediated neurotransmission is also altered in PD, and the involvement of different 5HT receptor subtypes is hypothesized (Huot et al., 2011). In invertebrates, 5HT, together with DA, are important neurotransmitters in locomotor activity during cilia movement (Katow et al., 2007; Sanderson et al., 1985) and 5HT is a main transmitter of the feeding system (Balog et al., 2012; Krajcs et al., 2014; Pirger et al., 2009). The chronic rotenone treatment decreased brain DA and 5HT levels and led to the progressive and irreversible decline in locomotion, feeding and in the life span of treated snails. All these observations corresponded well with earlier results where decreased feeding activity and DA level were observed on *Lymnaea* as a result of rotenone treatment (Vehovszky et al., 2007).

In both models, the decreased locomotor activity and monoamine levels correlated with quantitative changes of monoamine-metabolizing enzymes (S-COMT, MB-COMT and MAO-B). Our analysis was focused mainly on S-COMT and MB-COMT because MAO-B could not be identified in the CNS of snails, and according to the literature, it plays a minor role in eliminating monoamines (Sloley, 2004). Furthermore, the MAO-B level did not change in the rat 6-OHDA model either. Both COMT isoforms are widely distributed in the brain. COMT appears mostly as S-COMT and only a minor fraction is in the MB-COMT form. S-COMT is thought to be mainly responsible for the elimination of biologically active or toxic catechols, such as 6-OHDA (Mannisto and Kaakkola, 1999; Mulcahy et al., 2012; Roth, 1992). In our experiment, the S-COMT level significantly decreased in both the vertebrate and invertebrate toxin models. It is suggested that rotenone is metabolized mainly by oxidative processes like demethylation and after rotenone demethylation, monomethyl catechol and/or catechol forms will also appear in the brain. Therefore, S-COMT could also be involved in the elimination of rotenone metabolites (Haley, 1978). The decrease in S-COMT level observed in both of our models could be explained by changes in the original structure of S-COMT during enzymatic reaction (Mannisto and Kaakkola, 1999). We suggest that because of the structural changes of S-COMT, the anti-COMT antibody that we used may not recognize the active isoform of S-COMT in the WB experiments. At the same time, the MB-COMT enzyme plays a predominantly distinct role. It has been found that MB-COMT is primarily involved in the termination of DAergic synaptic neurotransmission when there are physiologically relevant low concentrations of catecholamines (Mannisto and Kaakkola, 1999). In our WB experiments the supernatant fraction was used; therefore, the amount of MB-COMT was very low in contrast to S-COMT in control situation in both models. Most MB-COMT activity resides in the sediment after centrifugation (Jeffery and Roth, 1984; Roth, 1992). However, following toxin treatment, a significant increase of the MB-COMT level was observed in both of our models. There are two possibilities to explain our results. First, the increasing concentration of MB-COMT could contribute to the decreasing DA level. Second, the MB-COMT level might increase in nervous tissues as a result of its release from disintegrating neurons. Treatment with 6-OHDA and rotenone increase the level of reactive oxygen species (ROS) in the cytoplasm, inducing cellular disintegration (Tsushima et al., 2012). As well as COMT, sulfotransferases also play key roles in the metabolism of monoamine transmitters, including DA metabolism in both invertebrates and vertebrates (Sloley, 2004). A few invertebrate cytosolic sulfotransferases have been characterized from Nematoda and Insecta with evolutionary conserved sequences (Hattori et al., 2006, 2007, 2008; Pichu et al., 2011), but unfortunately, we do not have sequence information about sulfotransferases in molluscs. Drug-metabolizing sulfotransferase enzymes have a role in the elimination of various environmental contaminants in mussels (Oshima et al., 1994; Janer et al., 2005). The molecular mass of sulfotransferases in ticks is reported to be the same as our most intense nonspecific immunopositive band at ∼36 kDa in our WB experiment. This band also shows a similar decrease in concentration as observed in S-COMT after rotenone treatment. Therefore, we speculate that this band might be a sulfotransferase-like protein in the snail CNS. However, we do not have any molecular or biochemical verification because the role of sulfotransferase-like proteins has not been investigated in a toxin-induced neurodegeneration model.

Ninety-five proteins were identified from 6-OHDA-treated rat SN but of the PD-relevant proteins, a marked decrease was detected only in the PARK7 chaperone protein level. PARK7 (DJ-1) belongs to the peptidase C56 family of proteins and it has multiple functions as an antioxidant, an oncogene and a molecular chaperone both in vertebrates and invertebrates. Earlier studies established three possible mechanisms by which PARK7 exerts neuroprotective effects. First, it is able to stabilize the NRF2 protein, the antioxidant transcriptional master regulator, thereby preventing apoptosis induced by oxidative stress (Clements et al., 2006). Second, it may inhibit the protein-associated splicing factor (PSF), which normally has a transcriptional-silencing activity that stimulates neuronal apoptosis (Xu et al., 2005). Third, the protein blocks aggregation of mutant α-synuclein, preventing the formation of Lewy bodies, which are an important hallmark of PD (Xu et al., 2005). Based on these roles, it could be suggested that the protein also plays a key role in neurotoxin-induced parkinsonian models. Although PARK7 has already been described in insects and planarians (Lavara-Culebras and Paricio, 2007; Tsushima et al., 2012), the presence of PARK7 or a PARK7-like protein in molluscs is shown here for the first time. Because it is generally accepted that PARK7 functions as an antioxidative agent, we examined its concentration after neurotoxin treatment. PARK7 protein levels were examined in both 6-OHDA-induced rat and rotenone-induced snail models. Independent of the model species or the used toxins, the level of PARK7 was significantly decreased. This decrease in both models correlate well with data obtained in other vertebrate and invertebrate studies (Miyama et al., 2011; Tsushima et al., 2012). The observations suggest that PARK7 may have conserved antioxidant and neuroprotective functions in animals and humans.

### Protective effect of PACAP in toxin-treated animals

The effect of the neuroprotective agent PACAP on the insecticide rotenone-induced survival rate of snails was tested. All rotenone-treated snails died by day 12, in contrast to the 50% mortality observed in the rotenone+PACAP-treated group, despite a slightly higher initial mortality. PACAP is also known as a survival-promoting peptide in vertebrate species, acting on divergent signal transduction pathways. For example, PACAP plays a pivotal role in immunity and inflammation, thereby prolonging the survival of mice with ileitis (Heimesaat et al., 2014) and rats in kidney ischemia-reperfusion models (Szakaly et al., 2008).

Earlier, we observed that PACAP-treated rats show significantly better performance in behavioural tests in 6-OHDA-induced neurodegenerative models. For example, they do not show signs of hypoactivity and improvement of asymmetric symptoms is faster than in control animals (Reglodi et al., 2004). PACAP was able to prevent severe behavioural effects of rotenone in snails. The spontaneous locomotor and sugar-induced feeding activities significantly decreased in the behavioural test of both rotenone and rotenone+PACAP groups compared with control animals. These results suggest that PACAP has a protective effect on locomotor and feeding activities as well as in survival in both models.

Treatment of toxin-exposed animals with PACAP counteracted the harmful effect of the toxin since it prevented the decrease of DA levels in both models. This observation correlates well with our earlier results, where PACAP treatment saved ∼50% of the DAergic neurons after 6-OHDA-treatment in the SN pars compacta of rats (Reglodi et al., 2004). Both toxins evoke a mitochondrial dysfunction in DAergic neurons through a combination of oxidative stress and injury to the mitochondrial respiratory system (Duty and Jenner, 2011). 6-OHDA is known to act through complex I or III inhibition while the target system of rotenone is complex I. Both toxins increase the level of ROS in mitochondria, causing an elevated cytochrome-c concentration in the cytoplasm. Cytochrome-c, in turn, increases the level of caspase-9 and caspase-3. This pathway could activate the apoptotic signal in neurons, causing cell death and leading to a decreased DA level in the nervous tissue. The PACAP-associated neuroprotection in 6-OHDA- and rotenone-induced cell death is initiated by activation of adenylate cyclase (AC) and protein kinase A (PKA), leading to a rapid inhibition of caspase-3 activation in DAergic neurons (Falluel-Morel et al., 2004). The inhibition of complex I and III leads to reduced neuronal ATP production, which can be compensated by PACAP through the AC system. In contrast to DA, PACAP treatment could not compensate for the decrease in 5HT level in either the vertebrate or the invertebrate model. The reason for this discrepancy is unknown at the moment. Various studies have described either increases or decreases of the 5HT level in models of PD, indicating that the 6-OHDA-induced rat model is not the best for studying the role of 5HT in PD (Mulcahy et al., 2012).

The level of S-COMT decreased significantly after application of each toxin and could not be restored by PACAP. In contrast to S-COMT, however, the increased MB-COMT level was successfully restored by PACAP to the control levels in both toxin groups. Based on these results, we conclude that PACAP has no direct effect on metabolizing enzymes but it does appear to have a general cell protective effect via MB-COMT. Consequently, more neurons survive in the presence of PACAP, these surviving neurons do not disintegrate and the level of MB-COMT will be low, as in the control.

A novel observation is that PACAP raised PARK7 chaperone protein levels up to the control level in our rat parkinsonian model. However, in the invertebrate toxin model, no effect of PACAP on PARK7 was apparent, in contrast to the ameliorated behavioural effects and actions on DA and related enzyme levels. We hypothesize that this discrepancy might be due to the use of different toxins in our models or possibly, different molecular mechanisms of the protective effect of PACAP. Consequently, it is suggested that 6-OHDA in rats and rotenone in snails could activate different ROS-producing signal transduction pathways.

In summary, we conclude that the neuroprotective effect of PACAP in different neurodegenerative animal models is well correlated with neurotransmitter levels. The monoamine and enzyme levels changed in parallel in 6-OHDA- and rotenone-induced models; the only difference was in the PARK7 content. It is believed that PACAP stimulates evolutionary conserved cellular mechanisms in both models, leading to neuroprotection. In conclusion, the rotenone and 6-OHDA rat and snail *in vivo* parkinsonian models successfully present some clinical properties of the disease and might offer an alternative way for studying the molecular mechanisms of the protective effect of PACAP. Finally, both 6-OHDA-induced vertebrate and rotenone-induced invertebrate parkinsonian models could also be used to investigate the neuroprotective effects of different polypeptides.

### Possible therapeutic potential of PACAP

The cytoprotective effects of PACAP have been confirmed outside the nervous system, including protective effects in the gastrointestinal, cardiovascular and urogenital systems. Therefore, possible future therapeutic approaches could be beneficial in several pathological conditions. However, based on the widespread occurrence of its receptors and the diverse actions of PACAP, side-effects of such treatments should also be considered. For example, as a vasodilator peptide, PACAP has been shown to lead to temporary decreases in blood pressure and facial flushing (Li et al., 2007; Seeliger et al., 2010), accompanied by edema and erythema when applied in humans (Seeliger et al., 2010), although other studies have found no alteration of blood pressure (Chiodera et al., 1995). Also associated with its potent vasomotor actions, PACAP is known to trigger migraine attacks in migraineurs (Schytz et al., 2009; Tajti et al., 2015). One study reported an increased heart rate after infusions in healthy volunteers (Birk et al., 2007). However, despite these side-effects, intravenous infusions are well tolerated (Li et al., 2007; Chiodera et al., 1995).

A lot of effort has been made to find analogues of PACAP peptide and/or specific PAC1 receptor agonists that are more stable, have long-lasting effects and preserve the protective functions without the vasomotor side-effects (Doan et al., 2011; Vaudry et al., 2010). A recent study has shown that a PACAP analogue could be well tolerated, is stable against cleavage by dipeptidyl peptidase and has strong neuroprotective effects (Lamine et al., 2016). Infusion of the analogue led to falls of mean arterial pressure in both PACAP- and Ac-[Phe(pI)(6), Nle(17)]PACAP(1-27)-treated mice, and both the intensity and duration of the pressure changes were reduced after injections of the analogue compared with the native polypeptide. This and similar approaches could serve as a basis for safe future therapeutic use of PACAP-induced neuroprotective pathways.

### Comparison of the PD models

The use of vertebrate (mouse, rat, and monkey) and invertebrate (snail, insect) animals to model PD allows us to study the disease mechanisms as well as to discover new drugs and possible treatments. Many models successfully reproduce features of the disease and key pathological properties of clinical PD within a shorter time (Cannon et al., 2009). For example, the hydrophilic 6-OHDA (induces oxidative stress triggered by ROS species via the vesicular DA and noradrenaline transporters) induces progressive neuronal degeneration leading to DAergic neuronal death and bradykinesia in less than 24 h or 1-3 weeks depending on the site of application (Jeon et al., 1995; Sauer and Oertel, 1994). The lipophilic rotenone (mitochondrial complex I toxin) also induces a progressive neuronal degeneration of DAergic neurons and bradykinesia in both vertebrates and invertebrates (Sherer et al., 2003; Srivastava and Panda, 2007). The rotenone model reproduces many features of PD, including the degeneration of DAergic neurons and intracellular inclusions. The reproducibility of this model is, however, difficult because of high mortality in both vertebrates and invertebrates (Jagmag et al., 2016; Vehovszky et al., 2007). Analysing behavioural data, monoamine levels and metabolizing enzymes in the two different parkinsonian models, we conclude that the vertebrate and invertebrate model are comparable, since similar changes were observed in behaviour, monoamine and enzyme levels. The only difference was obtained in PARK7 in the two models. Thus, the molecular mechanisms occurring in the PACAP and PARK7 interaction could only be examined in the vertebrate model. Therefore, the usefulness of the rotenone-induced parkinsonian model could be questionable on the proteomic level in invertebrate species. It could be a valuable parkinsonian model for investigating the molecular mechanisms causing the change in monoamine and enzyme levels, as well as for studying the mechanisms of neuroprotection. All toxin models have advantages and disadvantages and may extend our knowledge of the mechanisms of the disease and help us to discover possible treatments (Jagmag et al., 2016). In addition to the rat 6-OHDA parkinsonian model, here, we provide evidence for the usefulness of a cheap invertebrate *in vivo* parkinsonian model to test neuroprotective agents and explore their molecular mechanisms.

## MATERIALS AND METHODS

### Experimental animals

#### Snails

Pond snails (*Lymnaea stagnalis*) were bred at the Balaton Limnological Institute, MTA-CER, Tihany. Animals were kept in separate large holding tanks (30 litres) filled with filtered natural lake water from Lake Balaton (18-20°C). Snails were kept under a 12 h:12 h light:dark cycle and fed *ad libitum* with lettuce and a vegetable-based fish food (Tetra Werke Company, Germany). They were food deprived for 2 days before the beginning of the behavioural and biochemical experiments. Experimental animals were placed in separate tanks (10 animals per litre). All procedures on snails were performed in accordance with the Hungarian Council on Animal Care guidelines on the ethical use of animals (VE-I-001/01890-10/2013). Efforts were made to minimize both suffering and number of animals used in the experiments.

#### Rats

Wistar rats (weighing 200-250 g) were housed under standard laboratory conditions. Animals were maintained under a 12 h:12 h light:dark cycle with free access to food and water (Reglodi et al., 2004). All procedures were performed in accordance with the ethical guidelines approved by the University of Pécs (BA02/2000-15024/2011).

### Rotenone treatment in snails

We used a slightly modified published procedure (Vehovszky et al., 2007). Snails not older than 3-4 months (young animals) were divided into four groups (control, rotenone, rotenone+PACAP and PACAP). The control group of snails was kept in filtered natural lake water (Lake Balaton) and injected with 100 µl physiological solution. The rotenone group of snails was kept in a tank containing 0.5 µM rotenone (dissolved in dimethyl sulfoxide and added to the filtered natural lake water) and was injected with 100 µl physiological saline solution. The rotenone+PACAP group was kept in a tank containing 0.5 µM rotenone solution and was injected with 100 µl PACAP solution (100 µg in 1 ml physiological saline) for up to 12 days. In order to test whether PACAP alone had any effect on the survival of the animals, a fourth group was tested in the survival experiments. This PACAP control group was kept in filtered lake water and was injected with 100 µl PACAP solution (100 µg/ml). Since PACAP alone had no effect on survival, we used only the other three groups for further experiments. The whole survival experimental procedure was repeated four times (the total number of animals was *n*=40/group). For monoamine studies, 30 animals/group/experiment were used, whereas for behavioural tests (feeding and locomotion), WB and ELISA studies, 10 animals/group/experiment were used after 5 days of treatment. Snails were dissected after treatment and their CNS, containing 5 paired and 1 unpaired ganglia, was removed for analysis. Treatments were repeated daily in each group and water of both types was also changed every day. In all cases, physiological saline or PACAP solution was injected into the body cavity of snails using Hamilton syringes with fine needles. Because there is no blood-brain barrier in the snail (Sattelle and Lane, 1972), solution injected into the body cavity will have direct access to the CNS. The injection procedure was performed using previously published methods (Kemenes et al., 2002; Fulton et al., 2005).

### 6-OHDA treatment in rats

Rats were randomly divided into three groups (Reglodi et al., 2004). One group of animals was given 2 μl physiological saline, followed by 2 μl 6-OHDA (*n*=27) (Sigma) dissolved in physiological saline at a concentration of 5 μg/μl containing 0.2% ascorbic acid into the left SN. PACAP-treated animals (*n*=15) received 2 μg PACAP dissolved in 2 μl physiological saline as pre-treatment, followed by 6-OHDA lesion of the SN. A physiological saline-treated group served as a control (*n*=10). This group received only physiological saline in the same volumes (2+2 μl) as 6-OHDA- and PACAP-treated animals. Both injections were delivered into the left SN (5.5 mm posterior, 2 mm left, and 8 mm ventral from bregma point) with a Hamilton syringe over a period of 5 min, and the needle was left in place for another 5 min. Injection procedures were applied to all groups on day 1. All operations were performed under isoflurane anaesthesia.

### Behaviour tests

#### Locomotion

Snails from control, rotenone and rotenone+PACAP groups were individually placed in an experimental tank (10×20×3 cm; Salanki et al., 2003) on day 5 of treatment. After acclimatization for 10 min, the locomotion route of snails was marked continuously by a marker for 3 min. Digital photographs of each animal were taken using a Nikon D5100 camera after the test. Based on individual pictures, the traces made by a single animal were measured (in cm) and analysed with Mousotron v.8.2 software (BlackSun; www.techspot.com/download).

#### Feeding

Feeding behaviour was followed by placing the snails individually into a Petri dish filled with 20% sucrose solution, which evokes feeding activity, i.e. rhythmic opening/closing movements of the mouth (Kemenes et al., 1986). The feeding experiment was made on day 5 of treatment. After acclimatization for 10 min, the evoked feeding rate was characterized by a counter blind for the treatment for 2 min (the number of bites/2 min). Data were processed in OriginPro8.

### Determination of monoamines by HPLC-MS

The whole CNS of snails and SN region from the treated and non-treated sides of rats were measured. For extraction of monoamines, acetonitrile was applied containing 0.1% formic acid and 0.01 mass/vol% dithiothreitol. In rats, the SN was dissected 1, 3, 7, 9, 12, 14 and 16 days after the operation, and DA content was determined by HPLC-MS. Tissues were homogenized after addition of 200 µl extracting solution and were analysed with a high-energy ultrasonicator UIS250V (Hielsher Ultrasound Technology, Teltow, Germany) for 6×10 s, applying ice-cooling between cycles. Samples were then vortex mixed and centrifuged (Heraeus Biofuge Pico, Thermo Fisher Scientific, Waltham, MA, USA) at 8000 ***g*** for 5 min. Supernatants were placed in a SpeedVac concentrator (Eppendorf Life Sciences, Hamburg, Germany) at room temperature. The resulting samples were dissolved fourfold in ultra-pure water containing 0.1% formic acid and loaded into autosampler vials for HPLC-MS measurements (Sarvari et al., 2014).

Analyses were performed with a complex Ultimate 3000 (Dionex, Sunnyvale, CA, USA) micro HPLC system equipped with a quaternary pump, a degasser and a QExactive UHR spectrometer (Thermo Fisher Scientific). Separations were performed on a Kinetex PFP column (100 mm×2.1 mm i.d.; particle size, 2.6 µm; Phenomenex, Torrance, CA, USA). The flow rate was 200 µl/min, the injection volume was 5 µl and the temperature was kept at 4°C in the autosampler and 40°C in the column compartment. Xcalibur (Thermo Fisher Scientific) software was used for controlling the instrument, data acquisition and spectrum evaluations. A gradient elution consisting of mobile phases A and B (A, 0.1% formic acid in ultra-pure water; B, 0.1% formic acid in acetonitrile) was applied for the chromatographic separation. The first 2 min were an isocratic period, the mixing ratio was 97%:3% A:B eluents. The eluent composition was changed for 60% B over the following 4 min. The column was washed with 60% B for 4 min and equilibrated to the initial conditions with 1 min linear gradient and an isocratic period of 4 min. A QExactive mass spectrometer equipped with a HESI source was used for mass detection. The ionization source was operated with an endplate potential of 3 kV in the positive ion mode. The following electrospray parameters were kept constant during the analysis: drying gas (N_2_) flow, 10 l/min; auxiliary gas flow, 2 l/min; sheath gas flow, 2 l/min; capillary temperature, 320°C; S-lens RF level, 20%.

The minimum resolution of the mass spectrometer was 70,000. Filters of SIM and MS2 modes were used for selective and sensitive detection of DA and 5HT. The most intense precursor-to-fragment transitions were used for quantitative analysis; for DA, 154.08→137.06 m/z; for 5HT, 177.10→160.08 m/z; 35 eV was applied as the normalized collision energy (Sárvári et al., 2014).

### Analysis of protein composition

To determine rat brain tissue protein composition a previously published protocol of sample preparation and one-dimensional SDS-PAGE was applied (Maasz et al., 2014). Briefly, brain samples were homogenized in 1 M Tris-HCl, pH 8, containing 0.5 M EDTA, 0.7 M β-mercaptoethanol and 10% sodium dodecyl sulphate (SDS) and resolved on a 12% SDS-polyacrylamide gel. All bands were excised from the gel with a razor blade, digested with trypsin and analysed with Waters nanoACQUITY ultra-performance liquid chromatography (Waters Corporation, Milford, MA, USA) coupled to a nanoESI MS system. Aliquots (5 µl) of the samples were injected and separated on a 1.7 µm BEH130 C18 analytical column (75 µm×100 mm) using gradient elution at a flow rate of 350 nl/min. The mobile phase was (A) aqueous formic acid solution (0.1%) with (B) acetonitrilic formic acid solution (0.1%). Initial setting was 3% acetonitrile (v/v), which was increased to 10% over 1 min, then increased to 40% in 15 min. The total run time was 30 min. The column temperature was set at 35°C. The temperature of the samples was 4°C. The nanoUPLC system was connected to a Bruker Amazon SL ion trap MS instrument (Bruker Daltonics, Bremen, Germany) coupled with a Captive Spray source. The instrument was controlled using Compass v.1.3 software (Bruker Daltonics). The mass spectrometer was operated in positive mode. The scanning mass to charge range was m/z 100-3000 at a 1 Hz acquisition rate. Nitrogen was used as nebulizer gas, gas pressure was set to 0.6 bar, drying gas flow was 4 l/min at 180°C and the capillary voltage was set to 3.8 kV. Each intensive peptide peak was fragmented and the completed data were processed with the DataAnalysis v.3.4 software (Bruker Daltonics). The identification of proteins was carried out by searching in the limited rat taxonomy database of the NCBI and SwissProt using Mascot v.2.4.1 (Matrix Science, London, UK). The search parameters allowed for one missed cleavage site and 80 ppm mass error for the MS and 0.3 Da for the MS/MS mode.

### Western blot assay

Proteins from rat SN and snail CNS were separated on a 12% SDS-polyacrylamide gel and transferred to nitrocellulose membranes. After blocking with 3% non-fat milk in Tris-buffered saline at 2 h, the membranes were probed with antibodies recognizing: β-actin (Sigma, SAB5500001, 1:5000 dilution), COMT (Sigma, C6995, 1:1000) and MAO-B (Sigma, AV43557, 1:1000 dilution) at 4°C overnight. Membranes were washed four times in Tris-buffered saline (pH 7.5) containing 0.2% Tween (TBST) prior to addition of goat anti-rabbit horseradish peroxidase-conjugated secondary antibody (1:3000 dilution). Then samples were washed four times for 5 min in TBST and the antibody-antigen complexes were visualized by chemiluminescence method on conventional films.

Densitometry analysis was performed using Fiji ImageJ processing software. Values were normalized relative to their corresponding β-actin bands used as an internal standard. The analysis was repeated three times with brain samples obtained from separate groups of animals.

### Sandwich ELISA

The whole CNS of snails and the SN region from the treated sides of rats were used for ELISA. Samples were homogenized in reagent diluent (2.5 mg tissue/100 µl) then centrifuged (Heraeus Biofuge Pico, Thermo Fisher Scientific) at 8000 ***g*** for 10 min at 4°C. The supernatants were collected and transferred to ultra-high recovery Eppendorf tubes.

Special 96-well microplates (DJ-1/PARK7 DuoSet ELISA Kit; R&D systems) were coated with 100 µl/well of capture antibody at 0.8 µg/ml in PBS solution at room temperature overnight. The plates were washed three times with buffer (0.05% Tween 20 in PBS, pH 7.2-7.4). Blocking was performed with reagent diluent (300 µl/well, 1% BSA in PBS, pH 7.2-7.4) and incubated at room temperature for 1 h. After washing, the DJ-1/PARK7 standards were added (0.313, 0.625, 1.25, 2.5, 5 ng/ml) to both snail CNS and rat SN samples (100 µl/well). A control was prepared with capture and detection antibody containing reagent diluent. Triplicates made on the plate for each sample were incubated at room temperature for 2 h. The washing protocol was repeated. Then 45 ng/ml detection antibody in reagent diluent (100 µl/well) was added and incubated at room temperature for 2 h, followed by an additional washing step. HRP (horseradish peroxidase)-conjugated detection antibody was added at 1:40 in reagent diluent (100 µl/well) and incubated at room temperature for 20 min in the dark. The bound HRP conjugate was detected by adding 3,3′,5,5′-tetramethylbenzidine (TMB) (Sigma, 100 µl/well) and incubated in the dark at room temperature for 20 min. The presence of immune complexes was detected by development of a blue colour and the enzymatic reaction was stopped by adding 100 µl concentrated H_2_SO_4_. Finally, the optical density was analysed with a microplate reader (PerkinElmer, Victor3 1420 multilabel counter) at 450 nm, and correction was made for absorbance at 550 nm due to imperfections in the plate.

### Statistics

Statistical analysis was carried out using SPSS v.20 (IBM, Budapest, Hungary). Kaplan–Meier survival test with TaroneWare comparison was made. Differences in the levels of DA and 5HT between the experimental groups (snail: control, rotenone and rotenone+PACAP group; rat: control, 6-OHDA and 6-OHDA+PACAP group) were analysed. Normality of the dataset was investigated using the Kolmogorov–Smirnov test, homogeneity of variances between groups was investigated using Levene statistics. Analysis of variance was performed with one-way ANOVA with Scheffe and Tukey or Tamhane *post hoc* tests. Differences were considered statistically significant at *P*<0.05. Results in the figures are shown as mean±s.e.m.
